# On site production of [^18^F]PSMA-1007 using different [^18^F]fluoride activities: practical, technical and economical impact

**DOI:** 10.1186/s41181-021-00150-z

**Published:** 2021-10-13

**Authors:** Costantina Maisto, Anna Morisco, Roberta de Marino, Elisabetta Squame, Valentina Porfidia, Laura D’Ambrosio, Daria Di Martino, Paolo Gaballo, Michela Aurilio, Monica Buonanno, Aureliana Esposito, Marco Raddi, Secondo Lastoria

**Affiliations:** Nuclear Medicine Division, Istituto Nazionale Tumori - IRCCS Fondazione G. Pascale, Naples, Italy

**Keywords:** [^18^F]fluoride, Time of beam, [^18^F]PSMA-1007, Radiochemical yield (RCY), Radiochemical purity (RCP), Stability

## Abstract

**Background:**

Prostate-specific membrane antigen is overexpressed in prostate cancer and it is considered a good target for positron emission tomography/computed tomography imaging of primary cancer and recurrent/metastatic disease, as well as for radioligand therapy. Different PSMA-analogues labeled with [^68^Ga]gallium have been investigated, showing excellent imaging properties; however, only small amounts can be produced for each radiolabeling. Recently, a [^18^F]fluoride labeled PSMA-inhibitor, [^18^F]PSMA-1007, has been introduced, and it has ensured large-scale productions, overcoming this limitation of [^68^Ga]PSMAs. In this study, PSMA-1007 has been labeled with low (A), medium (B) and high (C) starting activities of [^18^F]fluoride, in order to verify if radiochemical yield, radiochemical purity and stability of [^18^F]PSMA-1007 were affected. These parameters have been measured in sixty-five consecutive batches. In addition, the estimation of [^18^F]PSMA-1007 production costs is provided.

**Results:**

The radiochemical yield for low and medium activities of [^18^F]fluoride was 52%, while for the high one it decreased to 40%. The radiochemical purity was 99% for all three activities. [^18^F]PSMA-1007 did not show radiolysis up to 8 h after the end of synthesis, confirming that the radiopharmaceutical is stable and suitable to perform diagnostic studies in humans for a long period of time after the end of radiolabeling. Furthermore, radiochemical stability was demonstrated in fetal bovine serum at 4 °C and 37 °C for 120′*.*

**Conclusions:**

A starting activity of [^18^F]fluoride of 90 GBq (B) seems to be the best option enabling a final amount of about of 50 GBq of [^18^F]PSMA-1007, which is promising as it allows to: (a) perform a large number of scans, and/or (b) supply the radiopharmaceutical to any peripheral diagnostic centers in need.

## Introduction

Prostate cancer (PC) is the second most common diagnosed cancer in men and the major cause of mortality and morbidity worldwide (Eeles and Ni Raghallaigh 2018; Zhou et al. 2014; Culp et al. 2020).

Prostate-specific membrane antigen (PSMA) is a transmembrane protein and it is considered a valuable marker for PC. PSMA is upregulated in poorly differentiated, metastatic, and hormone-refractory PCs, being not or poorly expressed in inflammatory and/or benign prostatic tissues, as well as other normal organs (Umbricht et al. 2017). For these reasons PSMA has been considered an optimal target for PC, either for staging or follow-up purposes (Afshar-Oromieh et al. 2015), by Positron Emission Tomography/Computed Tomography (PET/CT).

Different PSMA radiopharmaceuticals are still of great interest (Cardinale et al. 2020). ^68^Ga-radiolabeled PSMA synthetic peptides showed excellent imaging properties and diagnostic accuracy, which make them superior to conventional imaging tests currently used in clinics such as CT and bone scan (Wurzer et al. 2020). The relevance, gained in clinical practice, reflects its ability in more accurately identifying non metastatic PC patients from metastatic ones, allowing to select the best therapeutic option. The significant drawback of using ^68^Ga-radiolabeled PSMA synthetic peptides is the reduced number of patients who can be imaged by PET/CT at once. Such limitation is due to the relatively low amount of [^68^Ga]gallium available after each elution from the generator and its short half-life (T_1/2_ = 68′). Recently, ^18^F-labeled PSMA targeted radiotracers have been introduced and they are increasingly being used in clinical practice since then (Zippel et al., 2020; Werner et al. 2020). For each radiosynthesis, a large amount of ^18^F-labeled PSMA can be produced and consequently a significant and higher number of patients may be studied than those with [^68^Ga]PSMA. The [^18^F]fluoride half-life (T_1/2_ = 109′) and larger productions by cyclotron might also allow providing the distribution to several PET centers that are not more than 4 h away from the producing facility, equipped with cyclotron and radiopharmacy (Shammi et al. 2019). Preliminary clinical reports have suggested that fluorinated PSMA offers other advantages: higher tumor-to-background ratios (Kesch et al. 2017) and low renal excretion (Giesel et al. 2017).

Fully automated production of [^18^F]PSMA-1007 was established using several, commercially available synthesizers (Cardinale et al. 2017; Naka et al. 2020; Katzschmann et al. 2021).

The purpose of this research is to find the best parameters to achieve the maximal amount of [^18^F]PSMA-1007 for each single batch, in order to supply the radioligand not only to on-site patients, but also to neighbouring PET centres. In this setting, we focused our study on the following key points: role of different starting activities of [^18^F]fluoride on the radiochemical yield (RCY) at end of synthesis (EOS), stability of the radiolabeled PSMA over a 8-h period of time and the analysis of the production costs for a nuclear medicine center with its own cyclotron/radiopharmacy facility.

The compliance of the final product and relative quality controls were defined only recently by the monograph on the Edition 10th, 2020, of the European Pharmacopeia.

## Materials and method

### Radiolabeling of [^18^F]PSMA-1007

[^18^F]fluoride was produced with a 18/9 MeV cyclotron (C18/9 MeV, IBA, Belgium) by irradiating a standard volume of 2.6 mL of [^18^O]H_2_O (≥ 98% of purity; Taiyo-Nippon Sanso Corporation, Japan). The parameters of the beam, such as time and target current, were adjusted according to the desired final activity.

Radiosyntheses of [^18^F]PSMA-1007 were performed using a fully automated radiosynthesizer (AllInOne 36, Trasis, Belgium; with 36 manifold actuators and a disposable fluid pathway) as was previously described (Shammi et al. 2019). The use of disposable cassettes ensures clean and reproducible operations throughout the whole radiolabeling. This system uses three purification cartridges: Quaternary Methylammonium (QMA), C18 extraction cartridge (ec) and a PS-H^+^. C18ec and PS-H^+^ cartridges, stacked one on top of the other, must be properly attached to the cassette.

The reagents used for each synthesis of [^18^F]PSMA-1007 are: PSMA-1007 precursor (GMP grade), dimethyl sulfoxide (DMSO) for precursor, ethanol, Phosphate Buffered Saline (PBS), Tetrabutylammonium Carbonate (TBA-HCO_3_) water/ethanol solution 0.075 M. These reagents were purchased as a single kit (ABX, Germany).

### Quality controls on [^18^F]PSMA-1007

High Performance Liquid Chromatography (HPLC) analyses were carried out using a LC 20AD Pump with a SPD-20AV UV/VIS detector (Shimadzu, Japan) equipped with a GABI radiometric detector (Raytest, Elysia, Germany). A 5 µm C18 300 Å, 250 × 4.6 mm column (Jupiter®, Phenomenex, Italy) was used with a flow rate of 1 mL/min and the following gradient (acetonitrile 0.1% Trifluoroacetic acid (TFA) as solvent A, water 0.1% TFA as solvent B): 100% A for 5′, 25% A and 75% B in 3′, the same gradient for 4′ and then 100% B in 3′. The UV/VIS detector was set at 220 nm and 254 nm.

Thin-layer chromatography (TLC), was performed on Alugram silica gel 60 (Mackerey-Nagel, GmbH & Co. KG, Germany) sheets as stationary phase, using as mobile phase a v/v mixture of 60% acetonitrile and 40% water, according to European Pharmacopoeia Edition 10th, 2020. TLCs were analyzed by a storage phosphor system (Cyclone Plus, Perkin Elmer, UK).

The assay of TBA, as possible contaminant impurity of the final product, was performed by iodine-stained TLC, as previously described (Kuntzsch et al. 2014); residual solvents (ethanol and DMSO) were quantified by gas-chromatography system (GC 2010 Plus, Shimadzu, Japan) equipped with a flame-ionization detector (FID) and a capillary column (Elite-1301, 6% cyanopropylphenyl 94% dimethyl polysiloxane, L 30 m, ID 0.53; Perkin Elmer, UK). The temperature of the split was 240 °C and the one of the FID 280 °C.

Radionuclidic purity was determined using a multi-channel analyzer (Mucha Star, Raytest, Elysia, Germany) and half-life measurement by means of a dose calibrator (Atomlab 500, Biodex, USA).

[^18^F]PSMA-1007 was also tested for bacterial endotoxin through kinetic chromogenic Limulus Amebocyte Lysate (LAL) method (Endosafe Nexgen-PTS, Charles River, USA).

The radiochemical stability of [^18^F]PSMA-1007 was tested using different starting amounts of radioactivity, to verify possible effects, if any, on the final product. Samples from product vial (at room temperature) were analyzed by radio-HPLC, to detect the presence of degradation impurities or free [^18^F]fluoride, at different times (2 h, 4 h, 6 h, 8 h) after EOS.

In vitro stability studies of [^18^F]PSMA-1007 were performed in Fetal Bovine Serum (FBS, Sigma-Aldrich, Germany) at 4 °C and 37 °C up to 2 h, to simulate blood stream conditions.

Different volumes of [^18^F]PSMA-1007 were added to FBS to reach a final volume of 1 mL, keeping a constant activity of 37.0 ± 3.7 MBq, and the radio-HPLC was performed at different times after incubation (5′, 30′, 60′, 120′) for both temperatures.

## Results

In a series of sixty-five consecutive syntheses of [^18^F]PSMA-1007 and relative quality controls we have evaluated the use of three different starting activities of [^18^F]fluoride, identified as: low (A, 55.91 ± 6.69 GBq), medium (B, 89.06 ± 4.02 GBq) and high activity (C, 162.38 ± 6.46 GBq). These activities of [^18^F]fluoride were obtained by monitoring both essential parameters of the cyclotron: time/length of the beam (20′, 30′ and 55′ respectively for A, B and C) and constant target current of 60 μA.

The final volume of [^18^F]PSMA-1007 at the EOS was of 19 mL, with a mean activity of 28.71 ± 0.56 GBq for A, 46.84 ± 2.54 GBq for B and 65.70 ± 4.23 GBq for C; it appeared clear, colorless, particle-free, sterile with a pH ranging between 5 and 7.

The radiolabeling procedure of [^18^F]PSMA-1007, that is fully automated, has been shown to be very reliable. In fact, the measured RCY at EOS, not corrected for decay and related to a synthesis time of about 40′, was similar for A (52.09 ± 7.10%) and B (52.66 ± 3.40%), while for C it decreased to 40.26 ± 1.76% (Table 1).

**Table 1:**
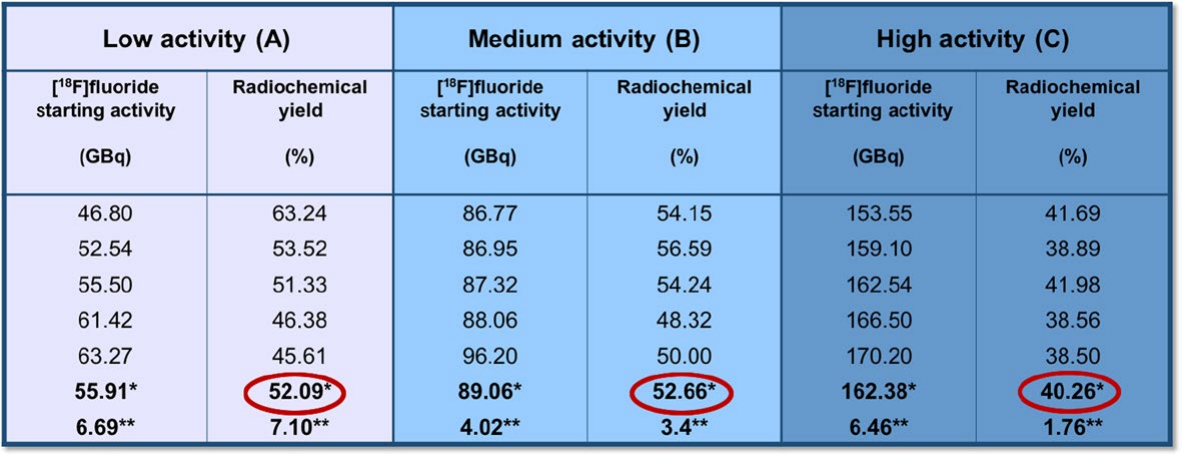
Comparison of [^18^F]fluoride (GBq) starting activity and related RCY( %) at EOS

RCY average values (%) estimated at EOS, not corrected for decay and related to a synthesis time of ≃ 40′. For activities A and B, RCY score is 52%, for activity C it decreases to 40%

^*^Average

^**^Standard deviation

Radiochemical purity (RCP), performed by radio-HPLC after each production, was ≥ 99% (Table 2); the retention time was ≃ 13.6′; no significant levels of free [^18^F]fluoride at EOS were detected; RCP, by TLC, was > 97% for the activities A and B, and 96.7% for C. Residual TBA was ≤ 2.6 μg/mL. The amount of ethanol was ≤ 10% v/v, the residual DMSO was ≤ 5000 ppm, according to European Pharmacopoeia method 2.4.24. The values of radionuclidic purity ranged between 490 and 531 keV for γ-spectrometry assay and between 105′ and 115′ for half-life measurement. The endotoxin value was less than 2.5 EU/mL.

**Table 2:**
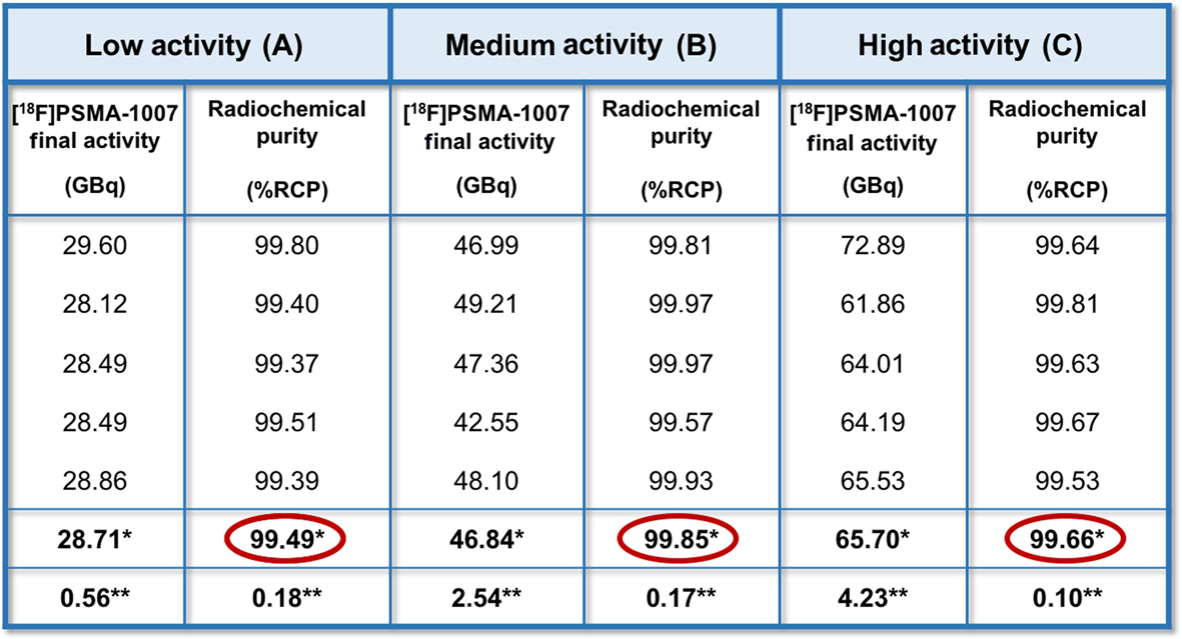
Comparison of produced [^18^F]PSMA-1007 activities (GBq) and related RCP(%)

In red, RCP average values (%) related to three activities (A, B and C) of the obtained [^18^F]PSMA-1007. RCP is > 99% and it is independent from the amount of final product at EOS

^*^Averages

^**^Standard deviation

**Fig. 1:**
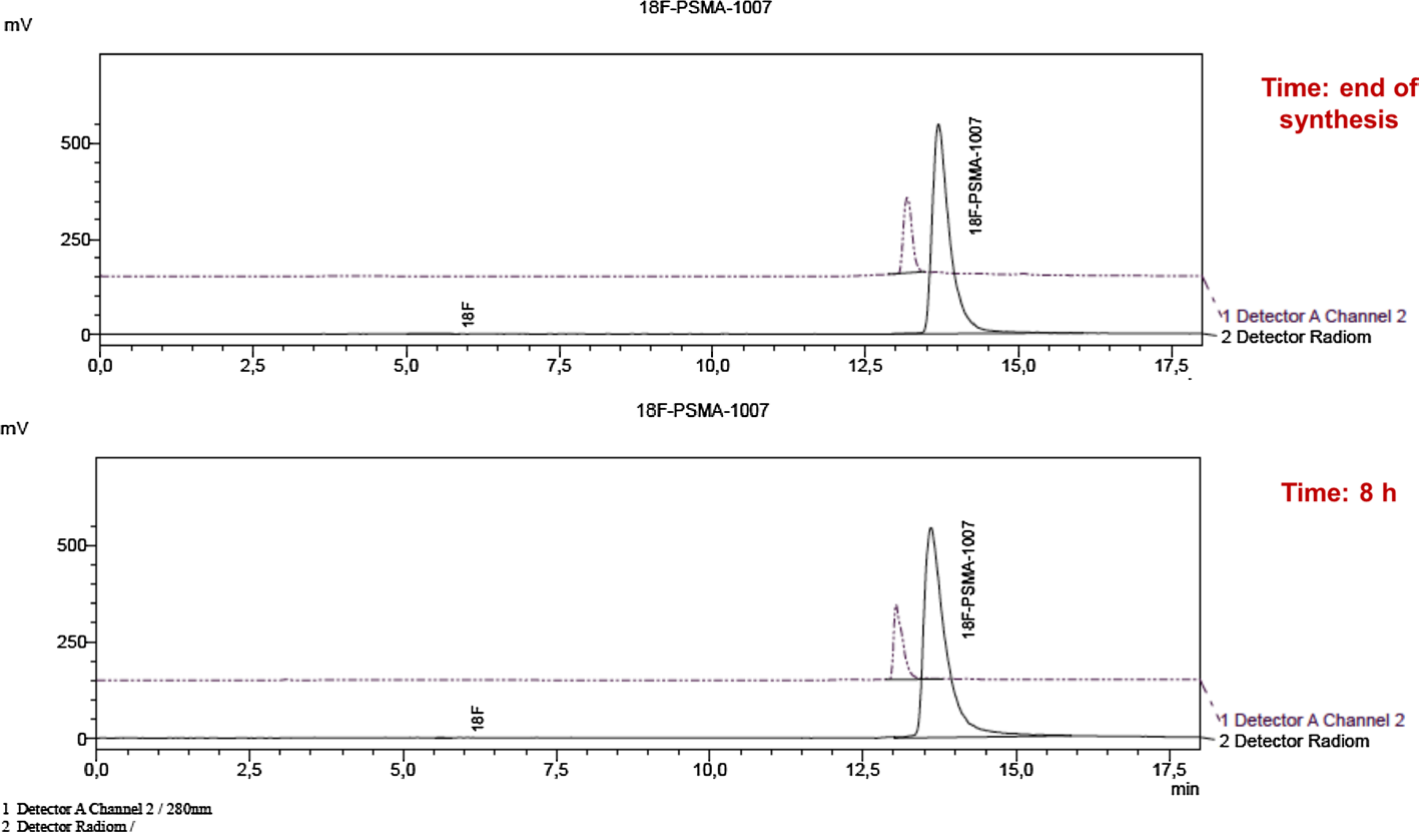
Radio-HPLC chromatograms performed at the EOS and after 8 h. The UV signal (Detector A Channel 2, 220 nm) of the PSMA-1007 and the radiometric of [^18^F]PSMA-1007 (Detector Radiom) due to the radiolabeled compound overlapped. The stability of radiolabeled peptide is confirmed up to 8 h after EOS

**Fig. 2:**
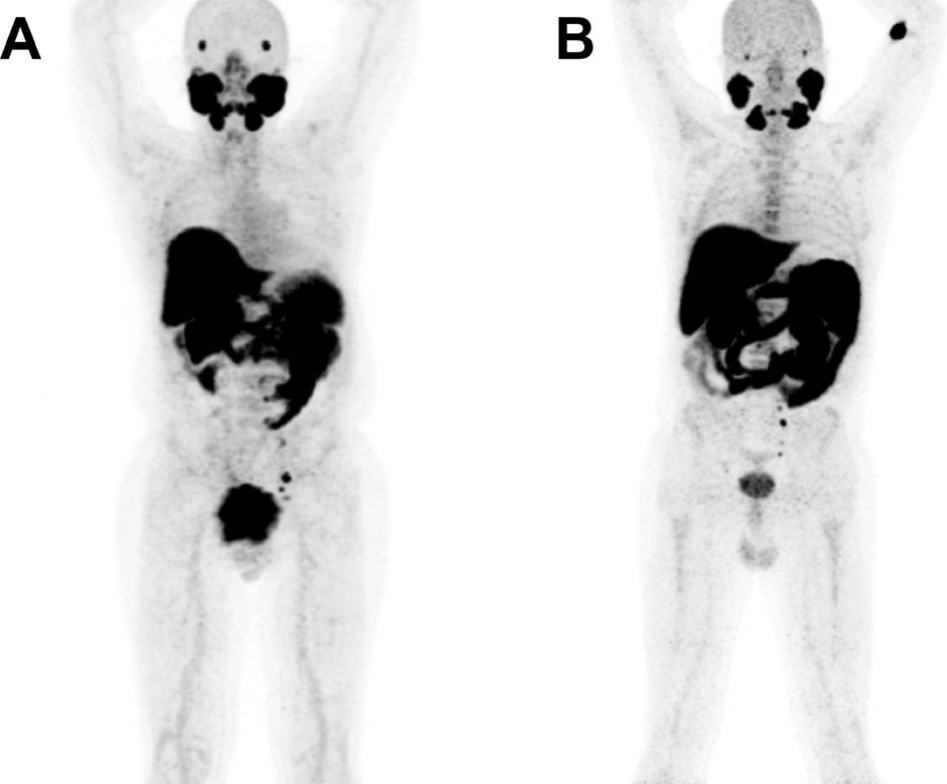
Example of different patients, with similar tumor burden, acquired on the same date, but receiving the injection at different times after the EOS. Patient A was injected about one hour after the [^18^F]PSMA-1007 EOS, and patient B six hours later

The radiochemical stability of [^18^F]PSMA-1007 was analyzed up to 8 h after EOS*,* and the radiochemical purity was > 99% with no evidence, on the chromatograms, of degradation impurities and detectable amounts of free [^18^F]fluoride (Figs. 1 and 2).

**Fig. 3:**
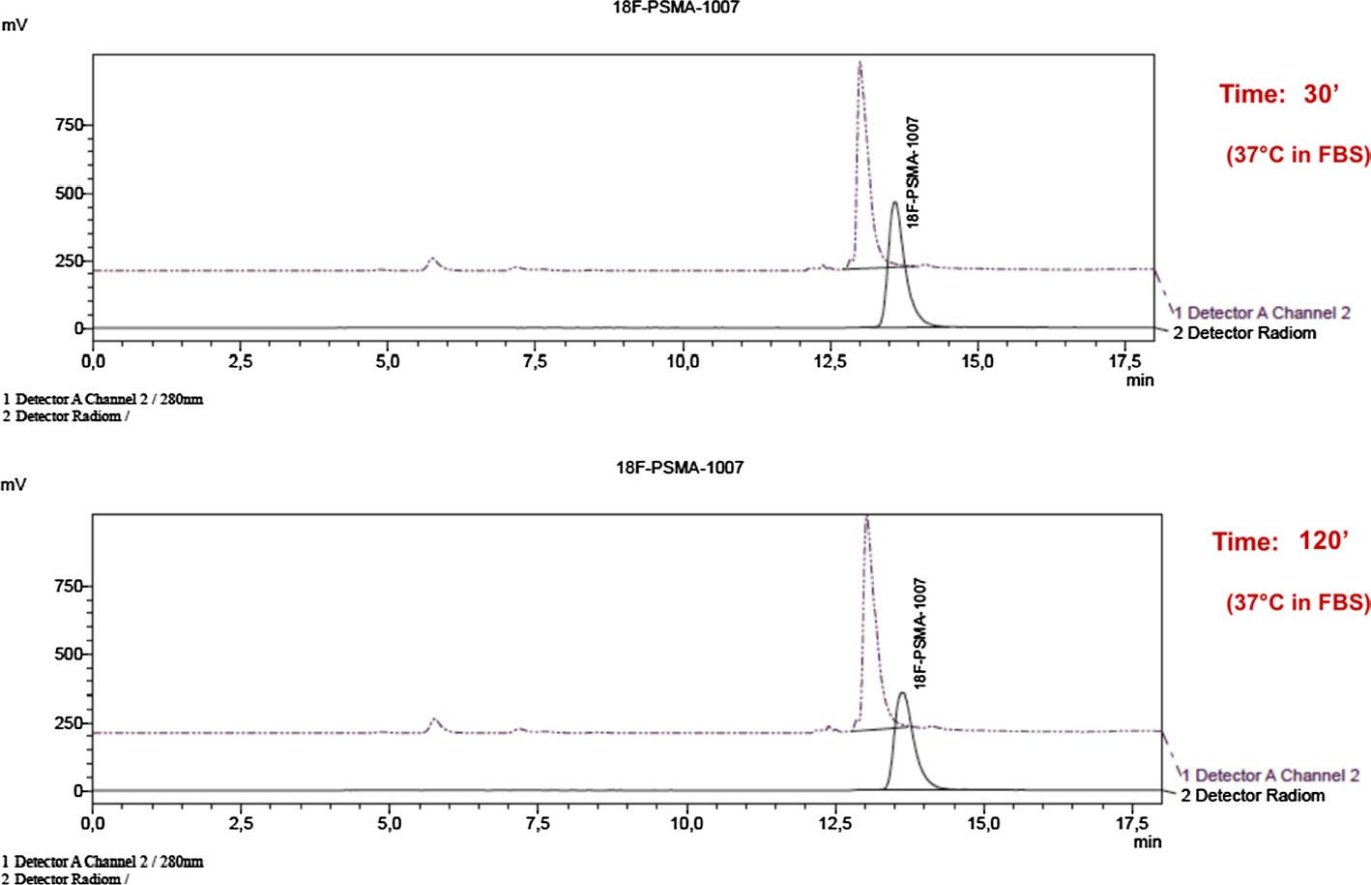
Studies of stability were performed by incubating of [^18^F]PSMA-1007 at 37 °C in FBS at the concentration of 37 MBq/mL. The chromatograms confirm the stability of the radiolabeled compound up to 120′

In order to verify radiochemical stability in vivo (to assure the integrity of the radiotracer in serum in the lapse of time between the somministration and the execution of the PET/CT exam, in terms of biodistribution to target site), the radiopharmaceutical was tested in FBS up to 120′, at 4 °C and 37 °C, showing absence of measurable radiolysis in both conditions, as reported by radio-HPLC analysis in Fig. 3. The results of these tests were further confirmed in patients administered and scanned within 2 h from EOS.

**Fig. 4:**
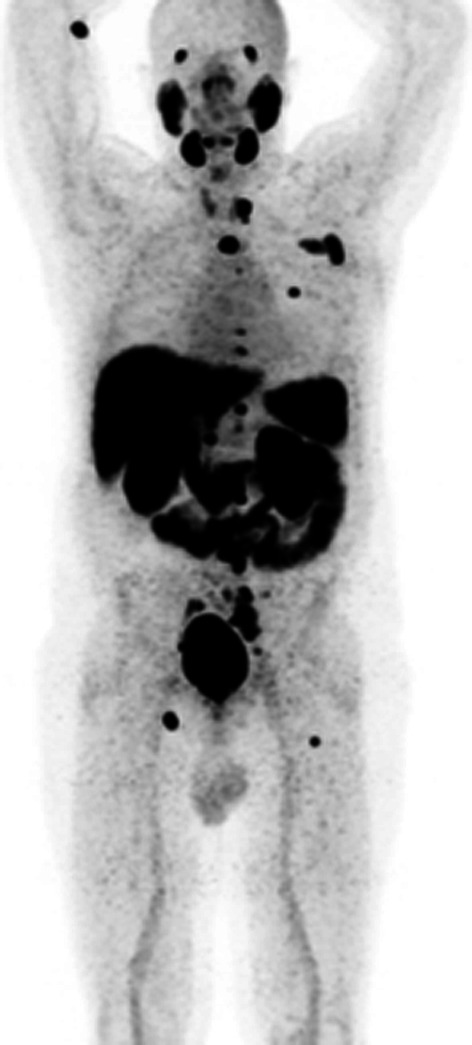
A patient with nodal and bone metastasis acquired after about 2 h from injection

No differences in the [^18^F]PSMA-1007 biodistribution, by PET/CT, were found in patients who received the radioligand at different times from EOS (Figs. 3 and 4), confirming that no degradation occurred by the time.

## Discussion

PSMA-PET/CT represents a ground-breaking diagnostic approach to stage and follow-up patients with recurrent PC. In particular, [^18^F]PSMA-1007 gained great interest for clinical applications, because of the physical decay advantages over [^68^Ga]PSMA, the opportunity to study a greater number of patients daily, the relatively low excretion by the urinary system and high tumor to background ratios (Sprute et al., 2021; Ioppolo et al., 2020). In addition, preliminary papers described elevated sensitivities also in small or tiny prostatic tumor lesions. A larger use of [^18^F]PSMA-1007 is expected for diagnostic purposes as alternative to [^68^Ga]PSMA-11 for the similar high diagnostic accuracy; furthermore [^18^F]PSMA-1007 shows a comparable biodistribution to [^177^Lu]lutetium radiolabeled PSMA-617, being the perfect option for selecting patients to treat by radioligand therapy.

Thus, the analysis to define and to optimize all possible variables in the process of [^18^F]PSMA-1007 radiosynthesis, may be useful for those who are planning to set up on site production. The labeling process to obtain [^18^F]PSMA-1007 is extremely reliable and it is characterized by elevated yield; radiolabeling failure (RCY 0%) occurred only in two cases and it was more likely related to mechanical errors within the module rather than to chemical failure during radiosynthesis. The flow of [^18^F]fluoride activity was monitored through the geigers of the synthesizer during the entire radiolabeling process.

In a case, at the beginning of the synthesis, 166.8 GBq were measured in QMA cartridge but 0 GBq were observed in the reaction vial. Similarly, in the second case, at the beginning of the synthesis, 125.7 GBq were measured in QMA cartridge but 0 GBq were observed in the reaction vial. The failure of QMA cartridge elution might be considered in both cases, in fact, investigations on the synthesizer at EOS revealed a defect of the eluent valve.

The trend of RCY and RCP, using three different activities of [^18^F]fluoride did not show significant differences. In details, RCY was 52% for batches labeled with activities A and B, resulting slightly higher than the average of [^18^F]PSMA-1007 yield, declared by module’s manufacturer: ≥ 40 ± 10% (not corrected for decay); whilst a RCY was reduced to 40% for batches labeled with activities C.

For each batch production reported, we evaluated the trend of critical steps of synthesis process that can be monitored by sensors: QMA cartridge and reactor (in the reactor it’s not possible to discriminate the activity due to free [^18^F]fluoride from the activity due to [^18^F]PSMA-1007). At the end, the final product activity was quantified only in the dose dispenser.

There are no significant differences of the trends on activity sensors for the three activities A, B and C. To quantify the loss of the residual radioactivity in the synthesis process, the critical components of the synthetic device (QMA, C18ec and PS-H^+^ cartridges, waste) were measured after 8 h from EOS. The activity detected on each of these components was respectively 0.37%, 1.48%, 16.7% and 12.3% of the [^18^F]fluoride starting activity. This justify only a part of the radioactivity loss.

The RCP values were substantially similar for all the three different starting activities of [^18^F]fluoride, showing values > 99% (99.49% for A; 99.85% for B; 99.66% for C). Moreover, stability studies were serially performed on [^18^F]PSMA-1007 in the period included from 2 to 8 h after EOS, showing no degradation of the radiolabeled PSMA.

According to these results, the medium starting activity of [^18^F]fluoride (B) coupled two advantages: a relatively short time of beam/radiolabeling (about 60′) and a better synthesis performance in terms of RCY (52.66 ± 3.4%) (Table 3). The increasing starting activity of [^18^F]fluoride is not directly proportional to the final [^18^F]PSMA-1007 amount. In fact, when the high starting activity C of [^18^F]fluoride was used for labeling PSMA, a decrease of RCY (40.26 ± 1.76%) along with an increase of the time of beam was observed (55′), without a significant gain in the amount of final available activity of [^18^F]PSMA-1007.

**Table 3:**
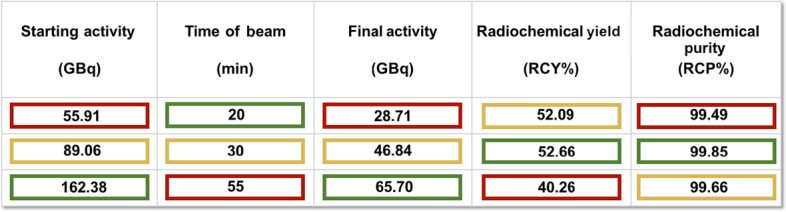
Parameters employed in the synthesis of [^18^F]PSMA-1007 for the three different activities

The table shows the variable parameters of the synthesis process: [^18^F]fluoride starting activity by cyclotron, time of beam, final [^18^F]PSMA-1007 activity, RCY and RCP. The best values of RCY and RCP were obtained for activity B, although the starting activity and the time of beam were not optimal. The best activity is B because it has no unfavorable conditions

Legend of colors:  unfavorable conditions,  optimal conditions,  intermediate conditions

Thus, in a referral institution for PC, with its own cyclotron/radiopharmacy facility, the option B is suggested to adopt, in order to produce a final amount of about 50 GBq of [^18^F]PSMA-1007. The stability of the radiotracer measured within 8 h after EOS and its constant binding ability, both justify and support the choice of using fluorinated PSMA for an entire working day, without the need of additional syntheses. Furthermore, these evidences justify the transfer of [^18^F]PSMA-1007 from radiopharmacy to PET centers located at distances covered in 3–4 h.

The starting amount of cold precursor of PSMA-1007 is fixed in 1.6 mg in commercially available kits used in our experience. Such amount is in excess, and it enables sufficient radioligand product to image prostate cancer lesions in humans, being not a limit for the clinical use, also varying the starting activities of [^18^F]fluoride.

In our site, the costs for the production of batches of 46.84 GBq of [^18^F]PSMA-1007 have been estimated in 5454€ (4.31€/37 MBq). These costs include the complete process, from cyclotron beam to dispensation of [^18^F]PSMA-1007, disposable/reagents, personnel, technologies and maintenance, radioactive waste dismission and general costs. On these bases, a reasonable price lower than 30 € for 37 MBq should be justified.

In the analysis of the costs, we accounted also those related to the wasting of the disposables used for the synthesis of [^18^F]PSMA-1007. In fact, they showed, in addition to [^18^F]fluoride, other radioactive contaminants with longer physical half-lives, including: [^58^Co]cobalt, [^57^Co]cobalt, [^51^Cr]chromium, [^54^Mn]manganese, etc. Thus, according to the current laws in European countries (Euratom BSS Council Directive 2013/59/Euratom), the removal and the wasting of these disposables follows very restricted rules. In fact, they cannot be eliminated very quickly, but they can be released after γ-ray spectrometry analysis and a prolonged stockage or released to authorized and specialized contractors.

## Conclusions

Methodological aspects for an optimal production of [^18^F]PSMA-1007 have been evaluated in order to define the best operating procedures. Suggested starting activity of [^18^F]fluoride should range from to 55 to 90 GBq, showing the following advantages:

- Short time of beam (30′).
- Highest radiochemical yield (> 50%) and purity (> 99%) at EOS.
- Relatively low costs of production with many available doses of [^18^F]PSMA-1007.

All these aspects strongly indicate that [^18^F]PSMA-1007 produced following this approach, may satisfy the growing requests of PSMA PET/CT in clinical practice, guaranteeing high quality radiopharmaceutical.

## Data Availability

Not applicable.

## Abbreviations

^18^F: [^18^F]fluoride
^68^Ga: [^68^Ga]gallium
C18ec: C18 extraction cartridge
CT: Computed tomography
DMSO: Dimethyl sulfoxide
EOS: End of synthesis
FBS: Fetal bovine serum
FID: Flame ionization detector
GC: Gas cromatography
LAL: Limulus amebocyte lysate
PBS: Phosphate buffered saline
PC: Prostate cancer
PET: Positron emission tomography
PSMA: Prostate specific membrane antigen
QMA: Quaternary methylammonium
RCY: Radiochemical yield
TBA-HCO_3_: Tetrabutylammonium carbonate
TFA: Trifluoroacetic acid
TLC: Thin layer chromatography
